# Supplementary material for: An evolutionary switch in ND2 enables Src kinase regulation of NMDA receptors
Source: Nat Commun. 2017 May 16;8:15220. doi: 10.1038/ncomms15220 (PMC5440837; doi:10.1038/ncomms15220)
Supplement: Supplementary Information — Supplementary Figures, Supplementary Tables, Supplementary Note, Supplementary References. [file ncomms15220-s1.pdf]

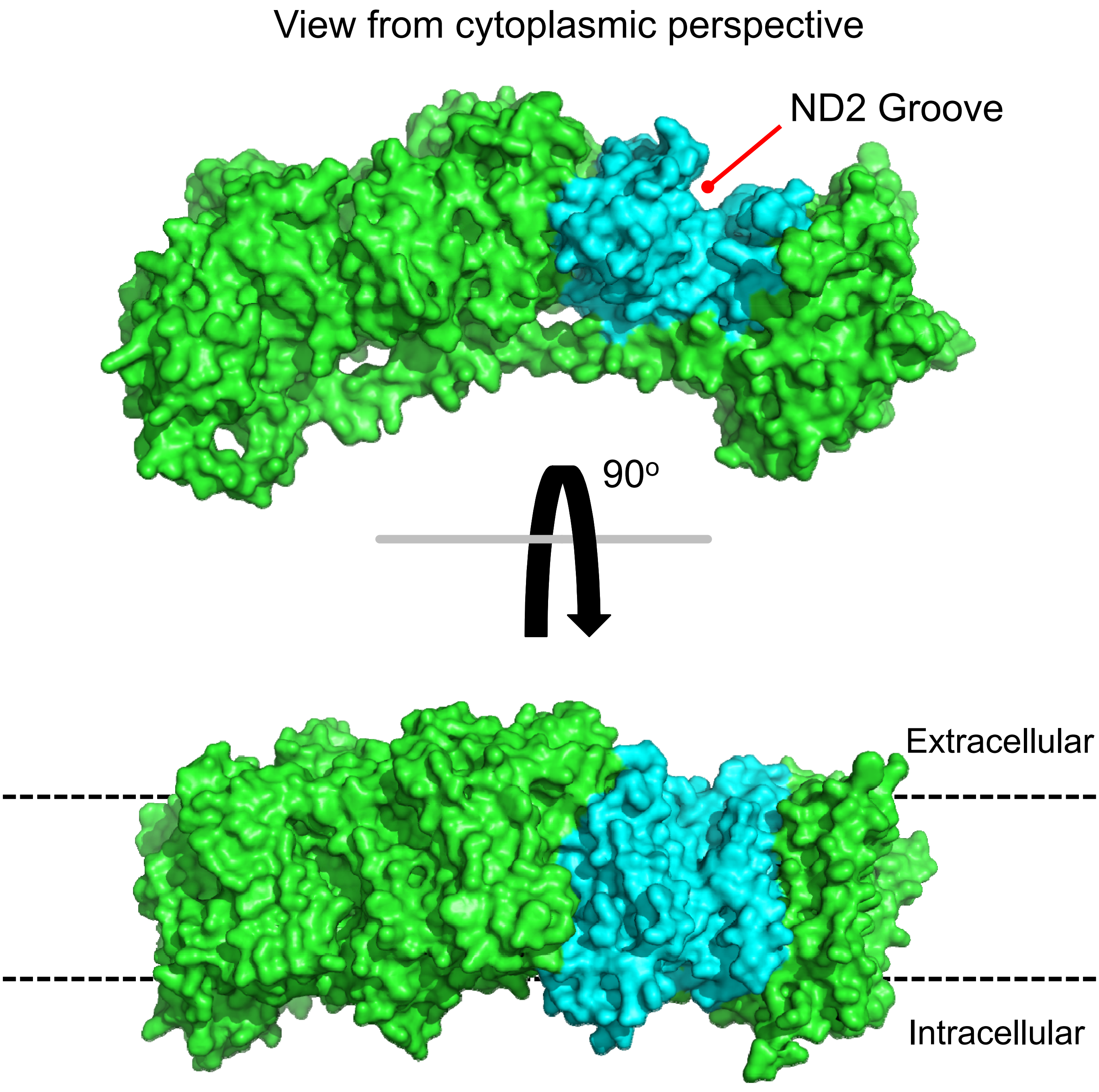

Supplementary Figure 1. Exposure of the ND2 groove within mitochondrial Complex I.
The membrane domain of complex I (green) with the ND2 subunit (cyan) laterally exposing its groove for a potential interaction with M4 of GluN1, shown in two orientations, from the cytoplasmic perspective (top) and 90° rotated (bottom).

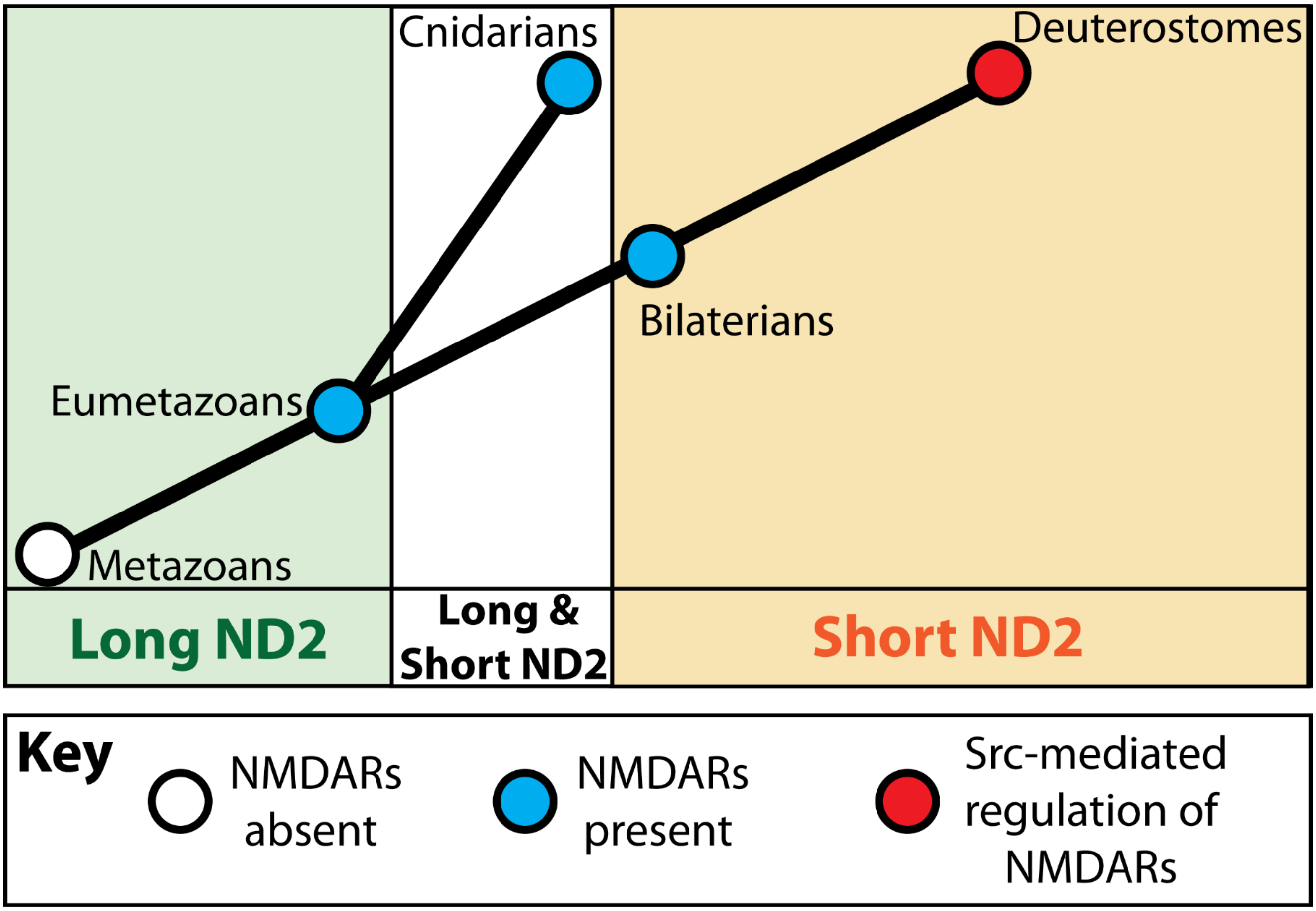

Supplementary Figure 2. Evolution of the ND2-NMDAR interaction. Evolution of the ND2 groove and the NMDAR subsequently enables ND2-mediated Src upregulation of the NMDAR. Cladogram describes the evolution of ND2 from 'long' (no groove) in early metazoans to 'short' (with groove) in bilaterians as well as the corresponding appearance and evolution of the NMDAR complex. Representative organisms from key phyla are depicted.

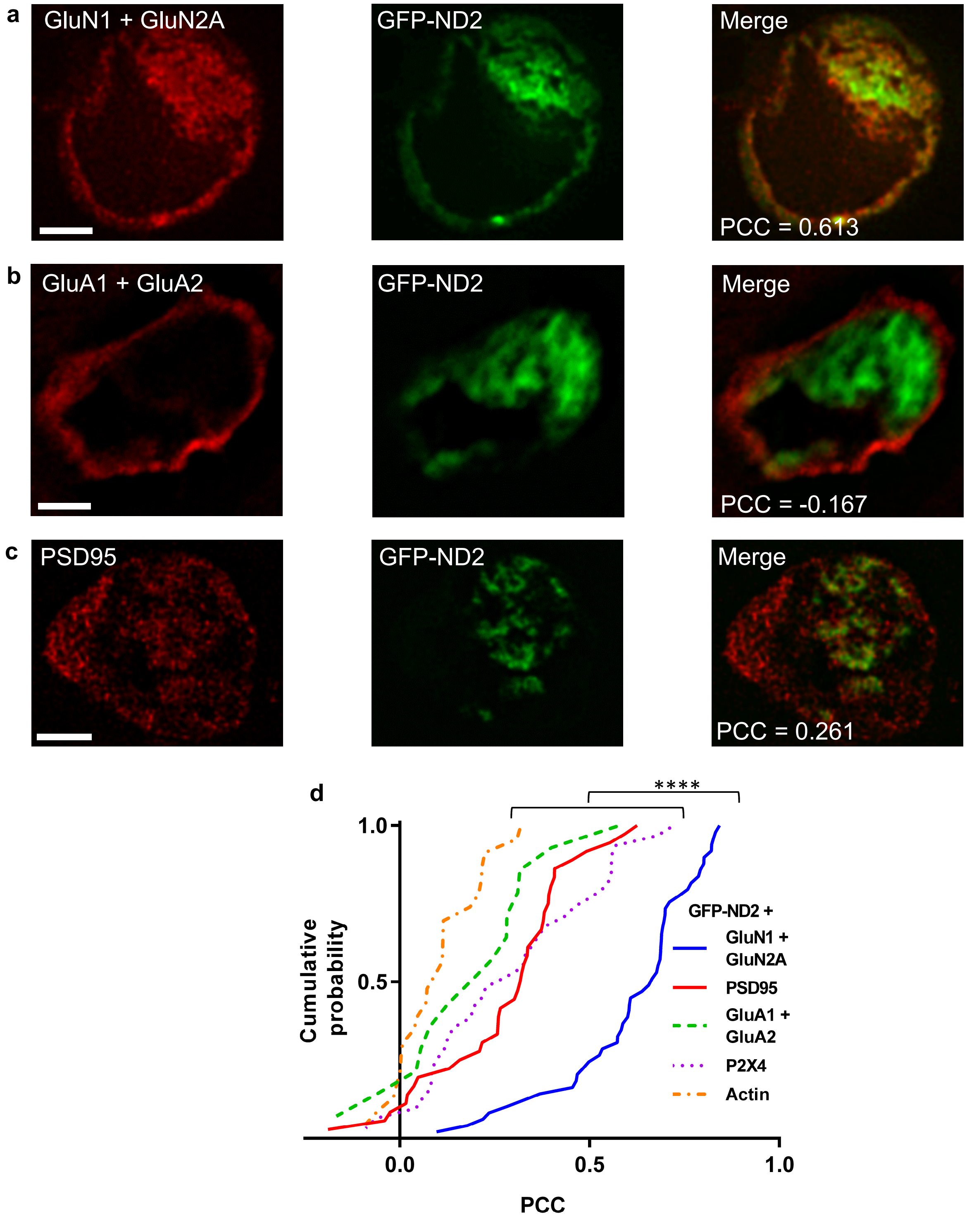

Supplementary Figure 3. ND2 interacts with NMDA receptors. (a) Representative images of HEK293 cells expressing GFP-ND2 with GluN1 + GluN2A, GluA1 + GluA2 (b), and PSD95 (c). (d) Cumulative frequency distribution of thresholded PCC values for GFP-ND2 with GluN1 + GluN2A, (mean PCC = 0.61 ± 0.03; n = 49), GluA1, (0.19 ± 0.05; n = 14), P2X4R, (0.30 ± 0.04; n = 31), PSD95, (0.28 ± 0.03; n = 36), and RFP-Actin, (0.10 ± 0.02; n = 23). Scale bars 3µm. Statistically significant differences between populations are indicated by the symbol '****' ($p<0.0001$), and were evaluated by Kruskal-Wallis non-parametric analysis of variance with Dunn's multiple post hoc comparison tests. n refers to number of HEK cells analysed. Results are presented as mean ± s.e.m.

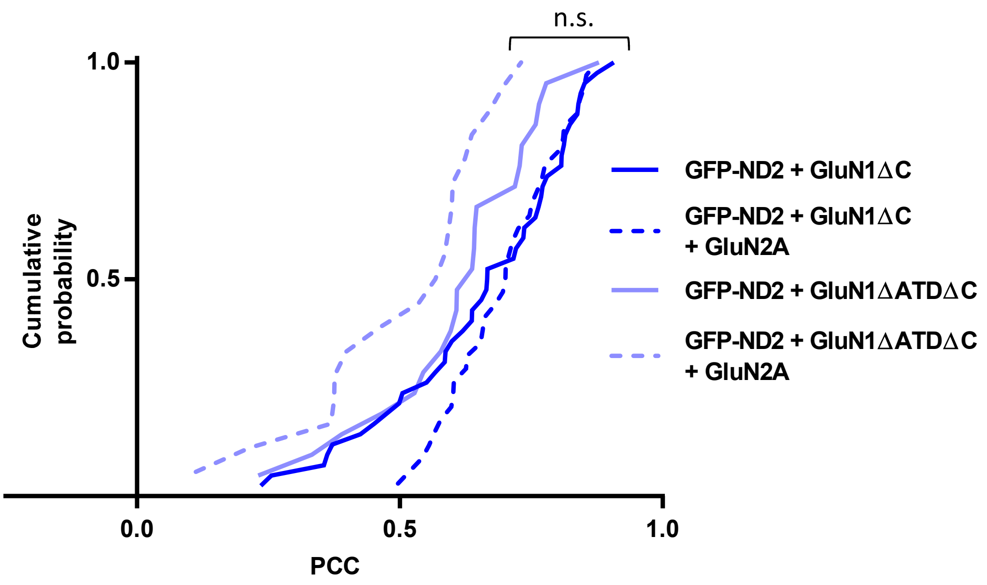

Supplementary Figure 4. GluN2A is not required for ND2-GluN1ΔC/ΔATDΔC interaction. Cumulative frequency distribution of thresholded PCC values for GFP-ND2 with GluN1ΔC alone, (mean PCC = 0.65 ± 0.03; n = 42), or + GluN2A (mean PCC = 0.70 ± 0.02; n = 34), and GluN1ΔATDΔC alone, (mean PCC = 0.61 ± 0.03; n = 21) or + GluN2A (mean PCC = 0.55 ± 0.03; n = 16). Cumulative probability distributions were evaluated by the Kruskal–Wallis test with Dunn's multiple post hoc comparison test. n refers to number of HEK cells analysed. Results are presented as mean ± s.e.m.

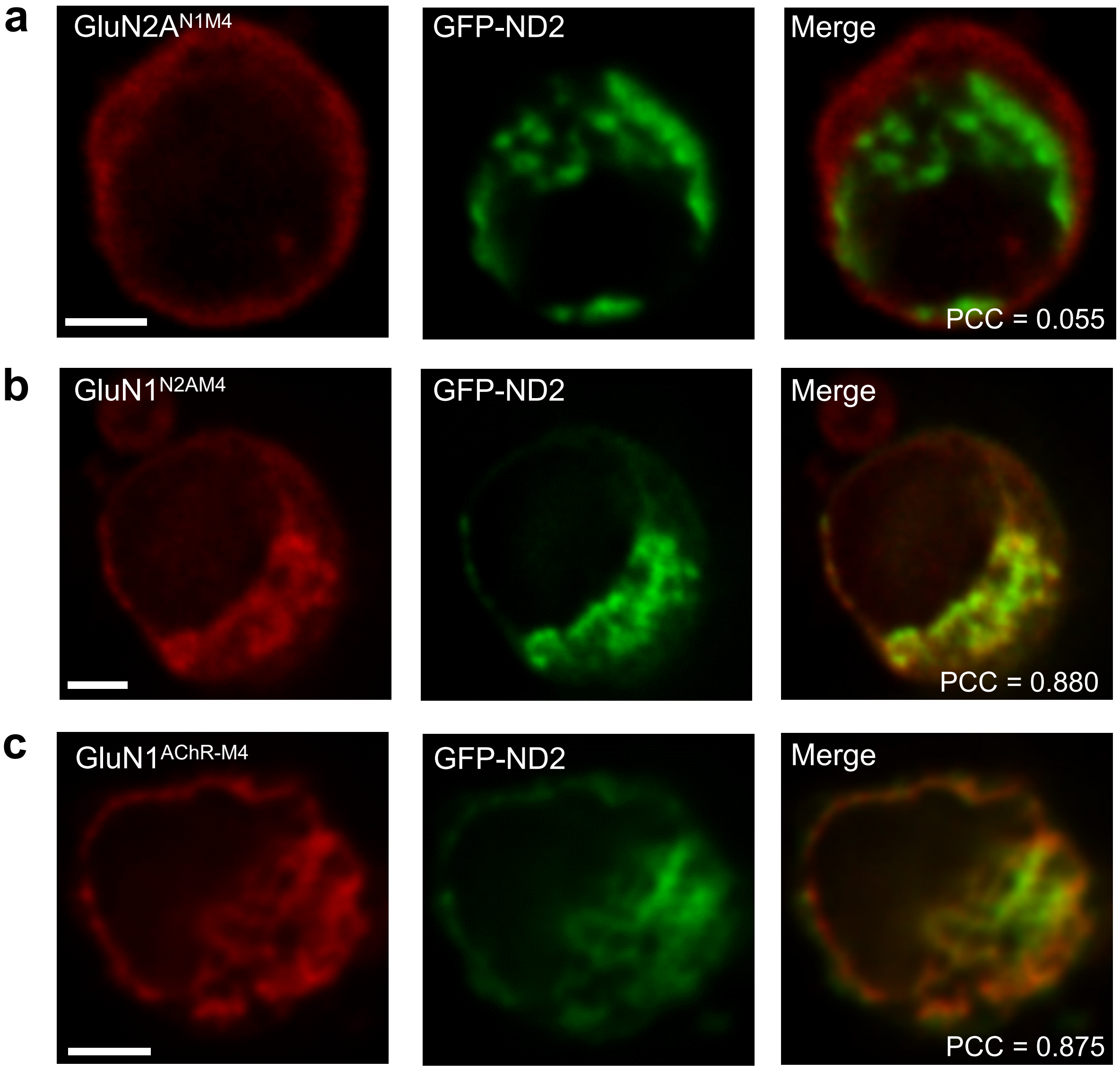

Supplementary Figure 5. ND2 interacts with GluN1 but not GluN2A M4 mutant constructs. (a) Representative images of HEK293 cells expressing GFP-ND2 with $\mathrm{GluN2A}^{\mathrm{N1M4}}$, $\mathrm{GluN1}^{\mathrm{N2AM4}}$ (b), and $\mathrm{GluN1}^{\mathrm{AChR\text{-}M4}}$ (c). Scale bars 3µm.

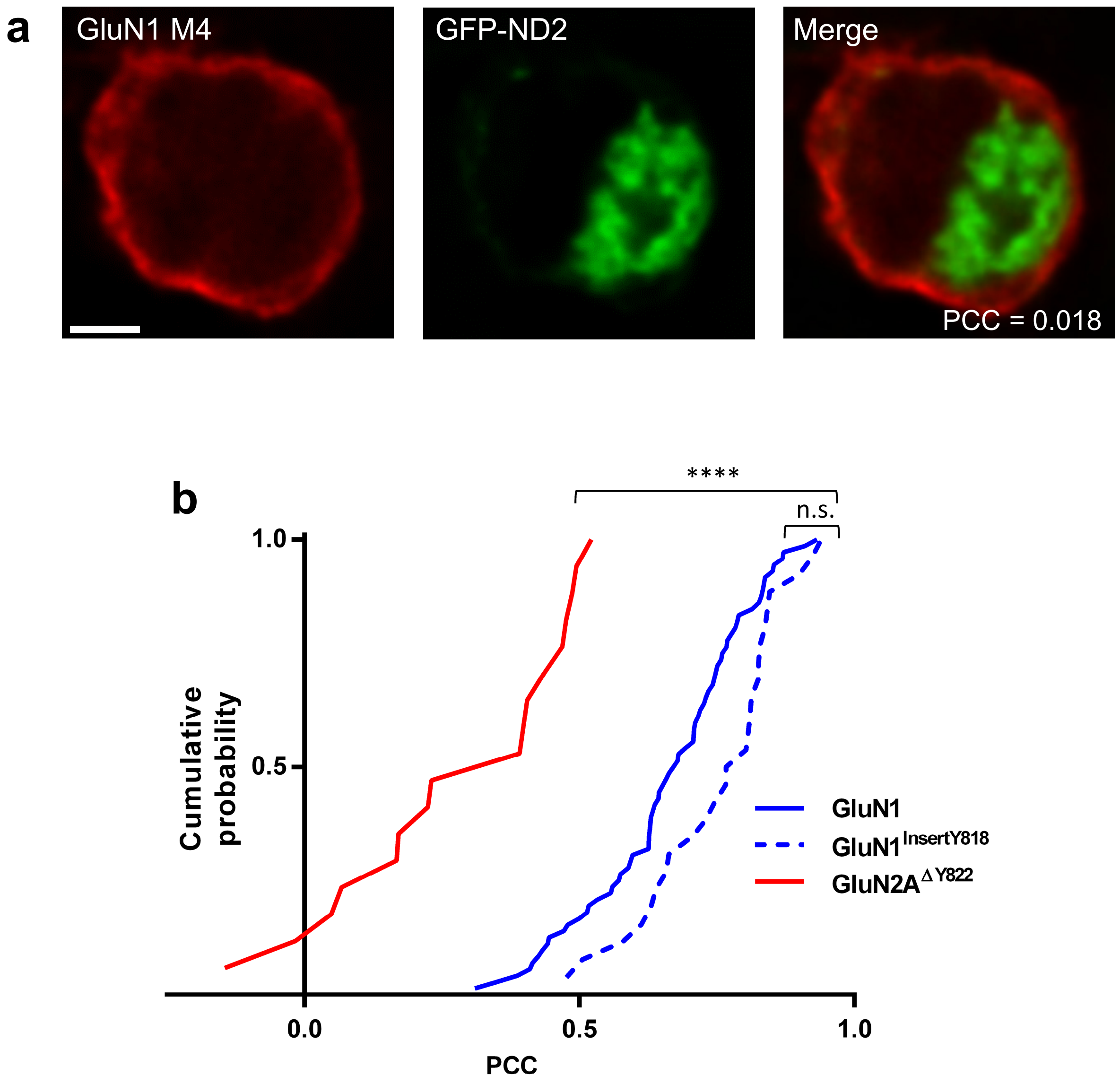

Supplementary Figure 6. ND2 interacts with GluN1^{InsertY818} but not with GluN2A$^{\Delta Y822}$
(a) Representative images of HEK293 cells expressing GFP-ND2 with GluN1 M4. (b) Cumulative frequency distribution of thresholded PCC values for GFP-ND2 with GluN1, (mean PCC = 0.78 ± 0.02; n = 29) GluN1^{InsertY818}, (0.75 ± 0.02; n = 26), and GluN2A$^{\Delta Y822}$, (PCC = 0.28 ± 0.05; n = 17). Scale bar 3µm. Statistically significant differences between populations are indicated by the symbol '****' ($p<0.0001$), and were evaluated by Kruskal-Wallis non-parametric analysis of variance with Dunn's multiple post hoc comparison tests. n refers to number of HEK cells analysed. Results are presented as mean ± s.e.m.

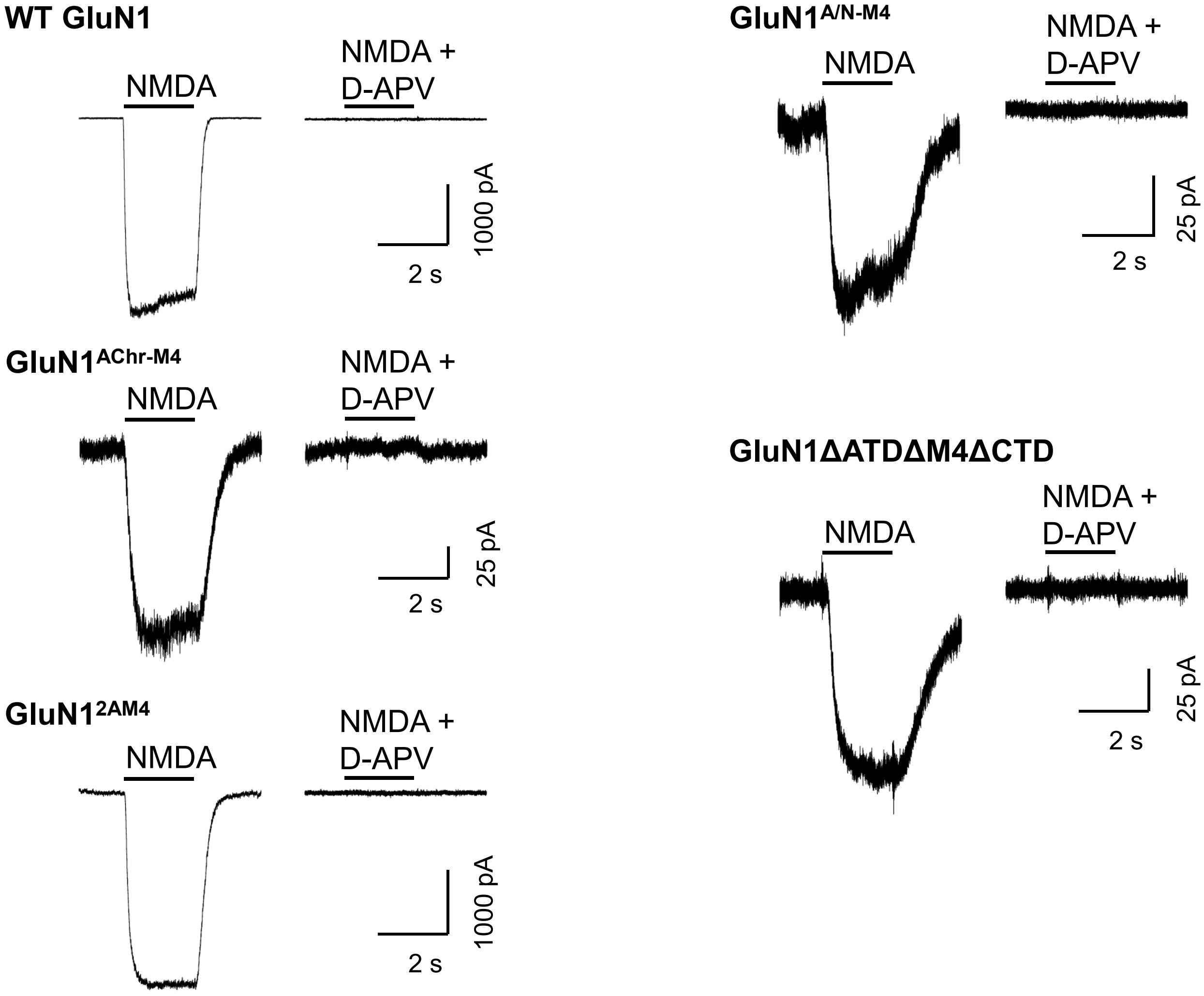

Supplementary Figure 7. NMDA evoked current in transfected HEK293 cells. Typical traces showing NMDAR currents evoked by NMDA (250 μM) in HEK293 cells transfected with GluN2A, PSD95 and GluN1. GFP was added to monitor the successfully transfected cells and only GFP positive cells were chosen for recordings. The mean evoked current amplitudes were -4478.5 ± 1591.0 pA for WT GluN1 (n = 5), -147.6 ± 103.3 pA for GluN1$^{A/N\text{-}M4}$ (n = 5), -68.7 ± 21.8 pA for GluN1$^{AChr\text{-}M4}$ (n = 5), -59.4 ± 31.4 pA for GluN1ΔATDΔM4ΔCTD (n = 4), and -3026.4 ± 1007.0 pA for GluN1^{2AM4} (n = 5). In each case currents were blocked by D-APV (250 μM). 10 μM glycine was present throughout the recordings. Results are presented as mean ± s.e.m.

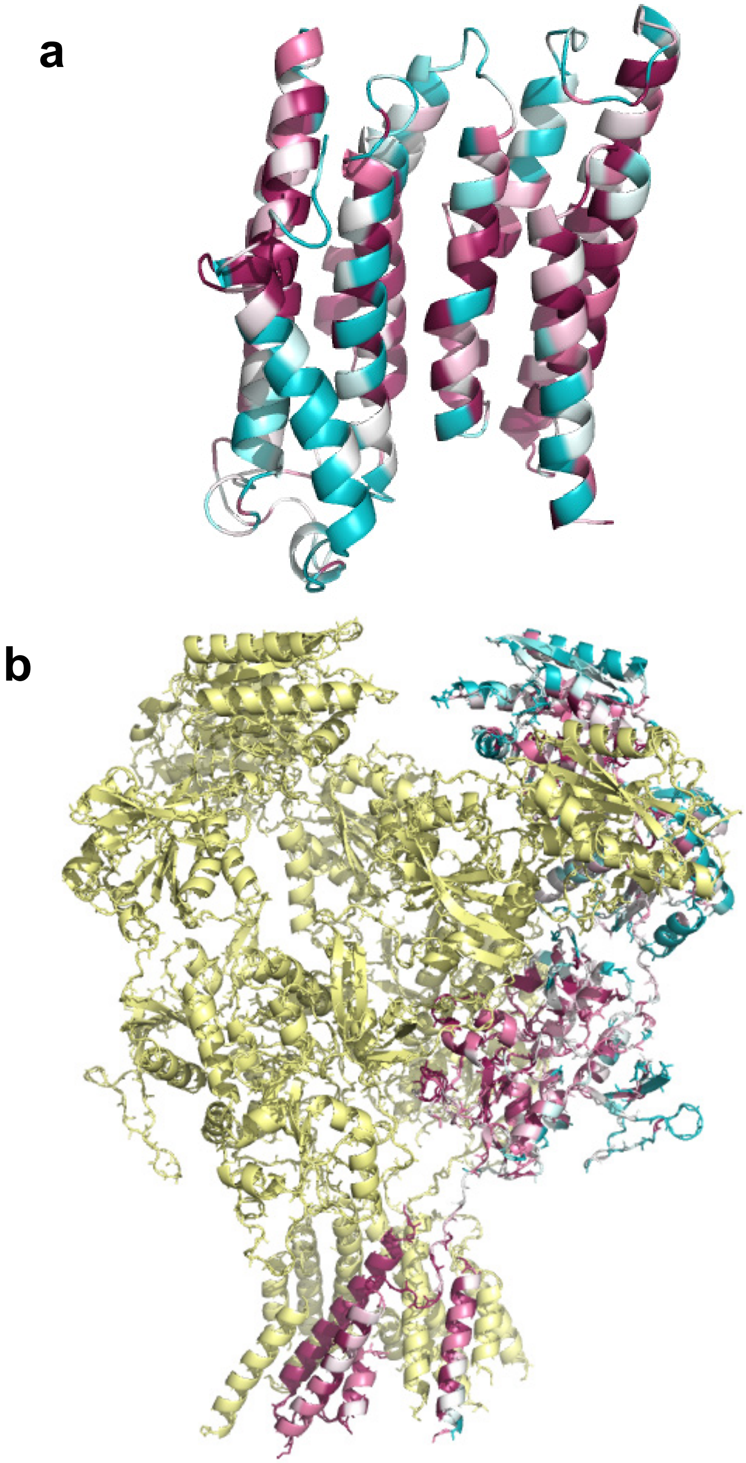

Supplementary Figure 8. Evolutionary conservation analysis of ND2 and NMDAR. Analysis of the the evolutionary conservation of amino acids in (a) ND2 and (b) NMDAR was performed to determine how conserved the NMDAR:ND2 binding interface is relative to the rest of the protein. We found no evidence that the binding interface is more highly conserved than the periphery. (a) For ND2, TMs 1 and 11, which line the outer rim of the ND2 groove, are among the least conserved helices while TM helices 2, 3 and 10, which are not part of the groove, are the most highly conserved. (b) For NMDAR, M2 and M3 helices, which form the channel, are the most conserved. These results agree well with our observations that the structural complementarity, rather than amino acid specificity, is critical. (Cyan = variable, Red = high conservation).

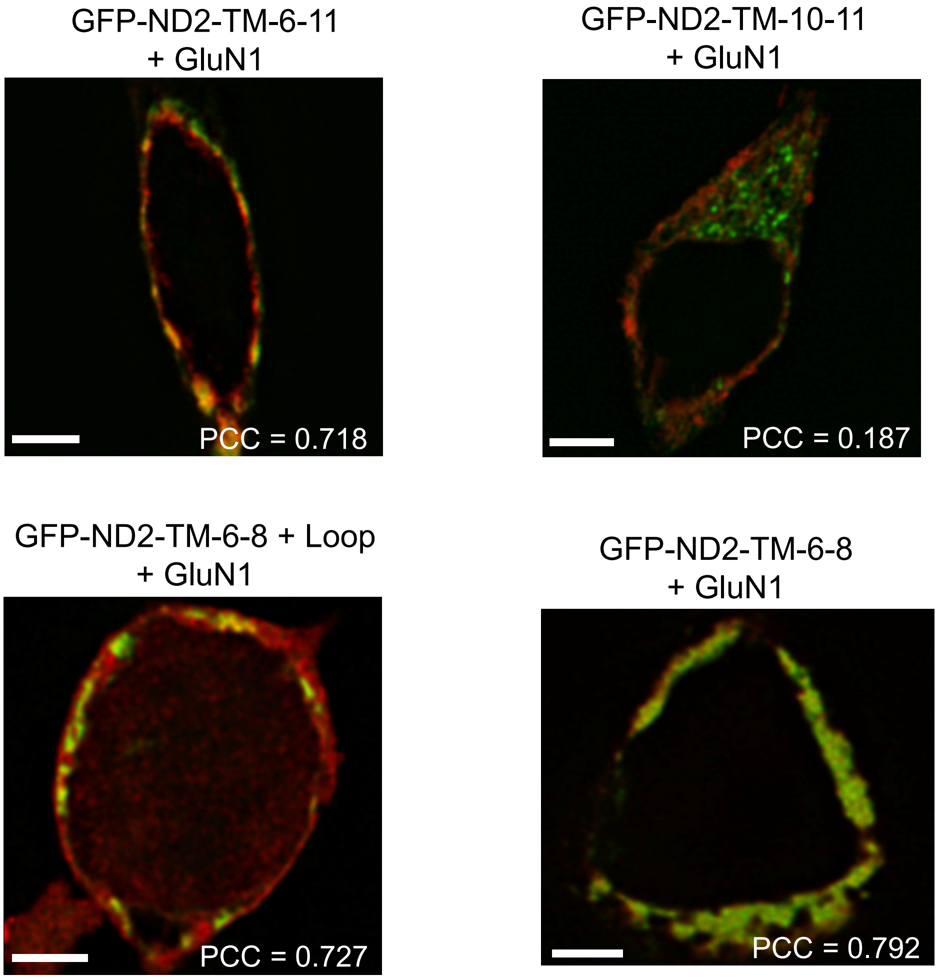

Supplementary Figure 9. GFP-ND2-TM-6-8 colocalizes with GluN1. Representative images of HEK293 cells expressing GluN1 + GFP-ND2-TM-6-11, GluN1 + GFP-ND2-TM-6-8 + cytoplasmic loop, GluN1 + GFP-ND2-TM-10-11 and GluN1 + GFP-ND2-TM-6-8. Scale bars 3μm.

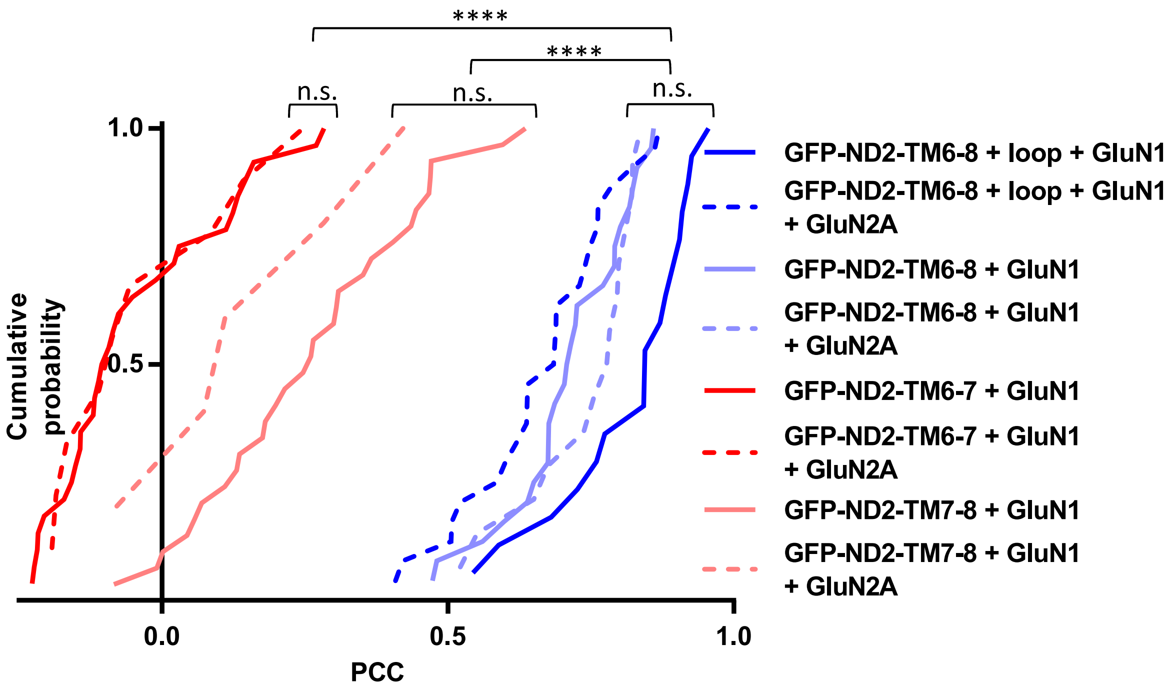

Supplementary Figure 10. GluN2A is not required for GluN1-ND2 fragment interaction. Cumulative frequency distribution of thresholded PCC values for GluN1 with GFP-ND2-TM-6-8 alone, (mean PCC = 0.71 ± 0.02; n = 24), or + GluN2A (0.74 ± 0.03; n = 14); GluN1 with GFP-ND2-TM-6-8 + loop alone, (0.75 ± 0.02; n = 30), or + GluN2A (0.66 ± 0.03; n = 34); GluN1 with GFP-ND2-TM-6-7 alone, (0.05 ± 0.03; n = 28), or + GluN2A (0.04 ± 0.05; n = 9); GluN1 with GFP-ND2-TM-7-8 alone, (0.26 ± 0.03; n = 29), or + GluN2A (0.16 ± 0.09; n = 5). Statistically significant differences between populations are indicated by the symbol '****' ($p<0.0001$), and were evaluated by Kruskal-Wallis non-parametric analysis of variance with Dunn's multiple post hoc comparison tests. n refers to number of HEK cells analysed. Results are presented as mean ± s.e.m.

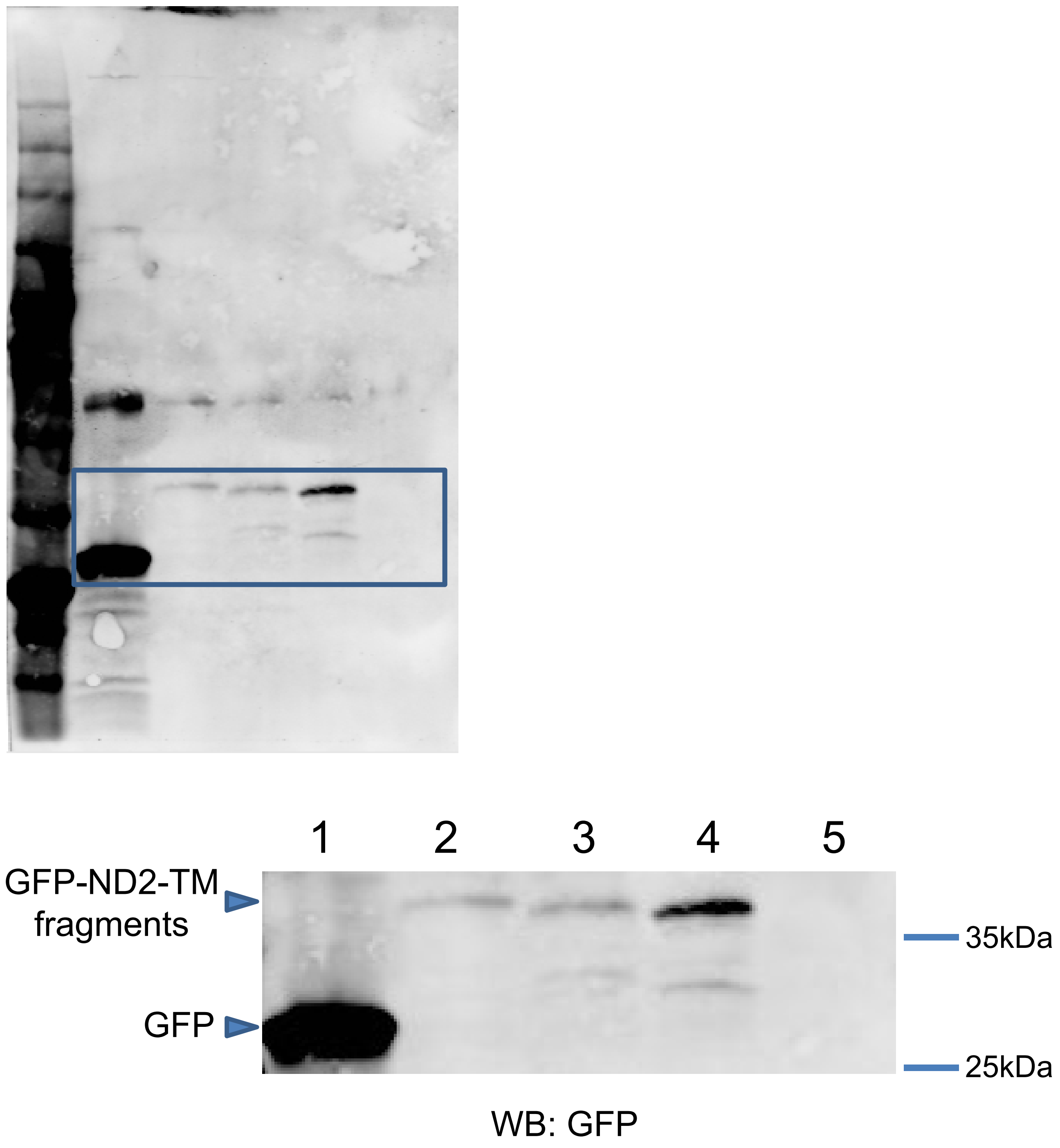

Supplementary Figure 11. GFP-ND2-TM fusion protein expression. Western blot analysis of HEK293 lysates transfected with 1 – GFP, 2 – GFP-ND2-TM-6-8, 3 - GFP-ND2-TM-6-7, 4 - GFP-ND2-TM-7-8, 5 – pcDNA3. 10 - 30ng protein was loaded to each well. GFP-ND2 fragments of the predicted molecular mass were detected. Uncropped blot included for reference.

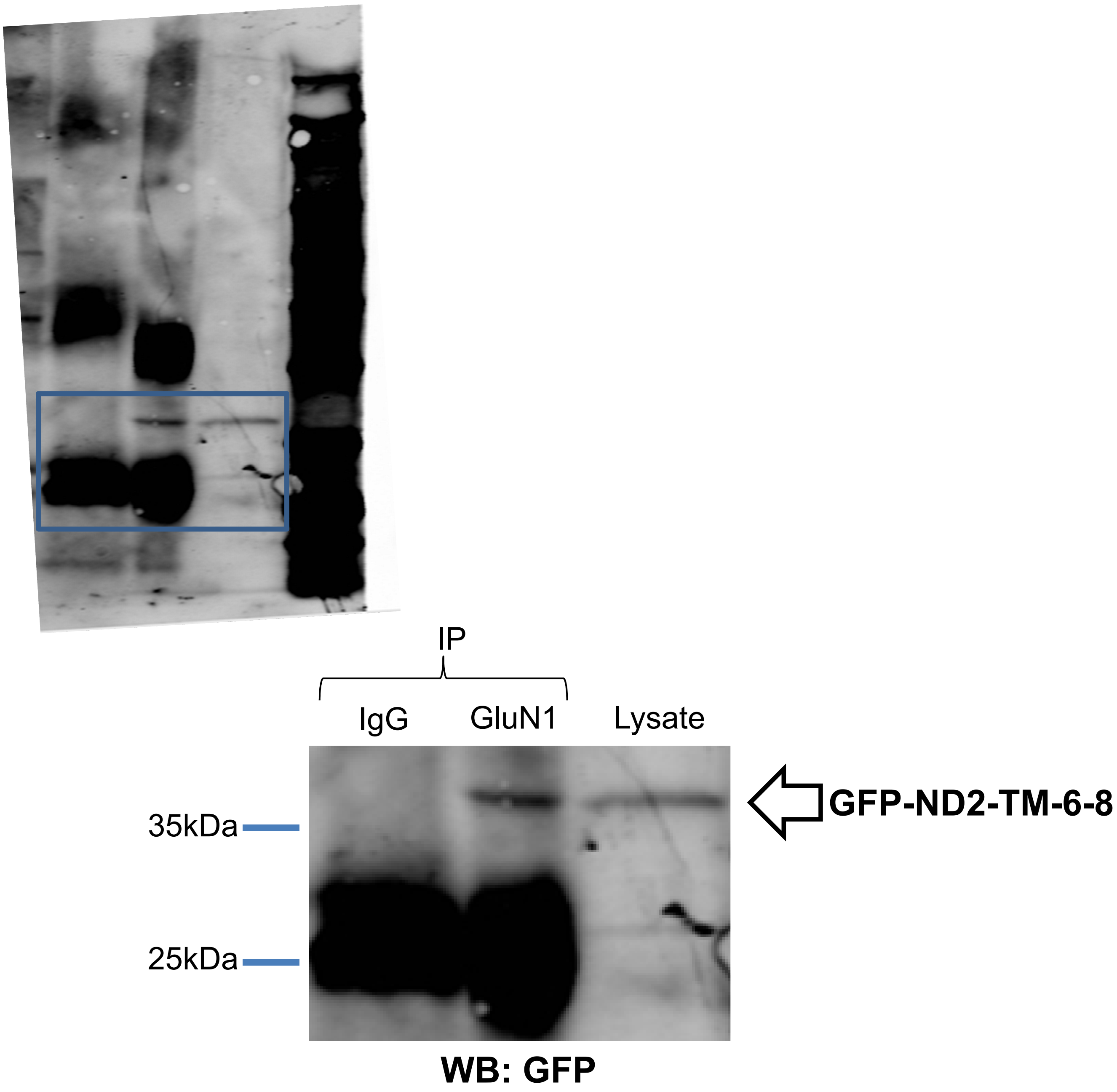

Supplementary Figure 12. GFP-ND2-TM-6-8 co-immunoprecipitates with GluN1. Lysates from HEK293 cells transfected with GFP-ND2-TM-6-8 + GluN1 were immunoprecipitated with anti-GluN1 antibody or non-specific IgG. Anti-GFP detected GFP-ND2-TM-6-8 by western blot in both the lysate and GluN1 immunoprecipitate but not in the non-specific IgG immunoprecipitate. Uncropped blot included for reference.

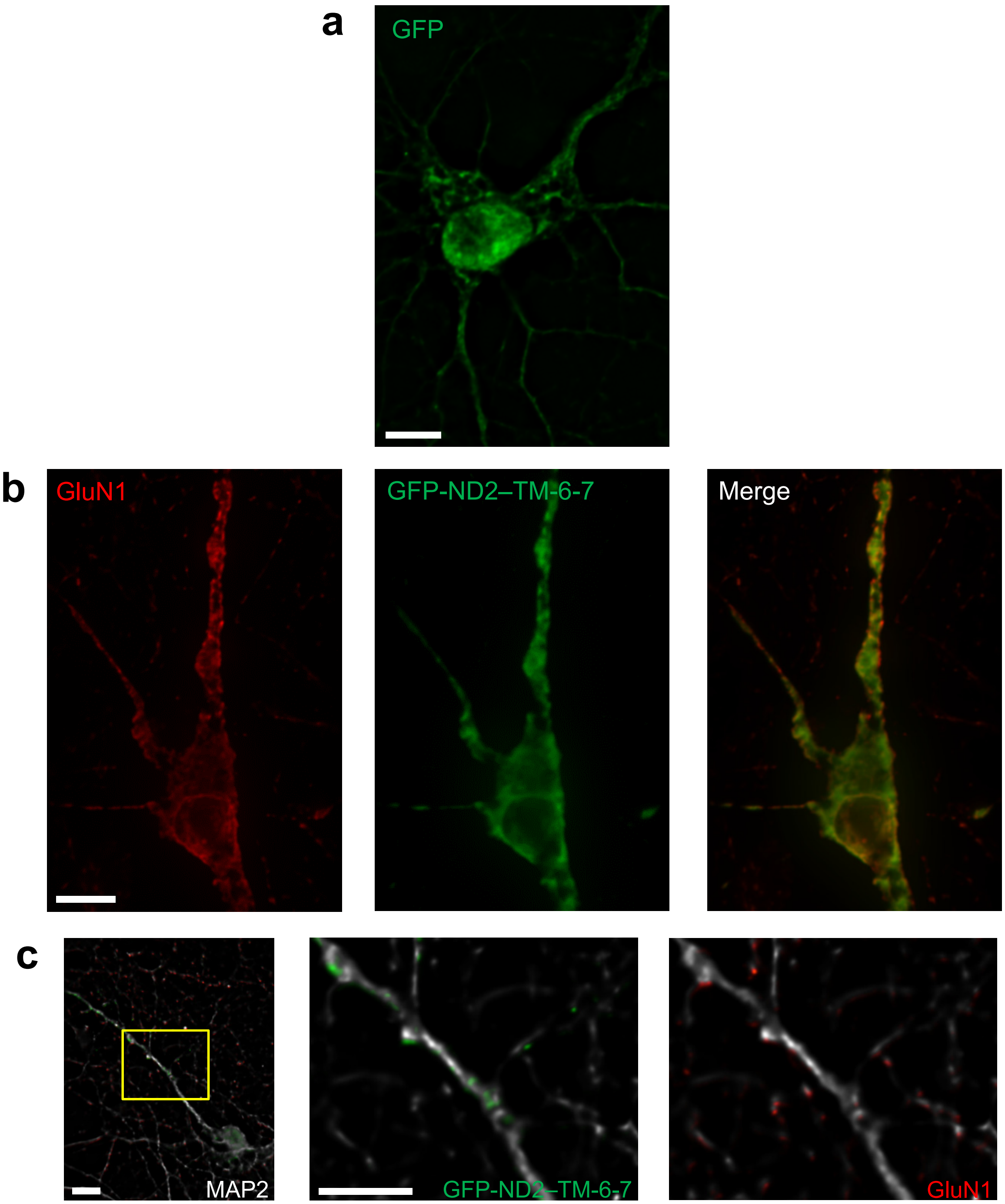

Supplementary Figure 13. Hippocampal neurons transfected with GFP/GFP-ND2-TM-6-7. (a) Immunocytochemically stained primary hippocampal neurons transfected with GFP alone or GFP-ND2-TM-6-7 (b,c). Anti GFP, GluN1 and MAP2 antibodies were used for visualization. Scale bars 10µm.

```
ND2       -------------------------------------------------------------------------------- 1
3RKO (L)  MNMLAL---TIILPLI----GFV-----LLAFSRGR--WSENVSAIVGVGSVGLAALVTAFIGVDF------FANGE--- 169
4HEA (T)  MALLG----TILLPLL----GFA-----LLGLFGKR--MREPLPGVLASGLVLASFLLGAGLLL----------SGG--- 152
4HE8 (I)  MTLAILAVFSVALTLL----GFV-----LPPQGVKR-------ATLL-----GLALALAS-L------------------ 120
3RKO (N)  MTITP----QNLIALLPLLIVGLTVVVVMLSIAWRRNHFL-----NATLSVIGLNAALV-SLWFVGQA------------ 159
3RKO (M)  MLLPW----LILIPFI----GGF-----L-CWQT--ERFGVKVPRWIALITMGLTLALSLQLWLQGGYSLTQSAGIPQWQ 176
4HE8 (M)  MVVLA-----VLLPVV----FGA-----LLLLGLPR------ALGVLGAG---LSFLLNLYLFLTH------PGGVA--- 139

ND2       ------------------------------------------------------ MNPLAQPVIYSTIFAGTLITALSSH 26
3RKO (L)  QTYSQPLWTWMSVGDFN IGFNLVLDGLSLTMLSVVTGVGFLIHMYASWYM-RGEE-GYSRFFAYTNLFIASMVVLVLADN 247
4HEA (T)  ARFQAE---WL--P--GIPFSLLLDNLSGFMLLIVTGVGFLIHVYAIGYM-GGDP-GYSRFFAYFNLFIAMMLTLVLADS 223
4HE8 (I)  ------LLTWGK--PFAFG-PYAVDGVSQVFTLL----ALLGALWTV--G-LVRS-GRFEFYLLVLYAALGMHLLASTRH 183
3RKO (N)  -------------GAMDVTPLMRVDGFAMLYTGLVLLASLATCTFAYPWLEGYND-NKDEFYLLVLIAALGGILLANANH 225
3RKO (M)  SEFDMP---WI--PRFGISIHLAIDGLSLLMVVLTGLLGVLAV-L-CSWKE-IEK-YQGFFHLNLMWILGGVIGVFLAID 247
4HE8 (M)  HAFQAP---LL--PGAGVYWAFGLDGLSALFFLTIALTVFLGAL--------VAR-VEGRFLGLALLMEGLLLGLFAARD 205

ND2       WFFTWVGLEMNMLAFIPVLTK-----KMNPRSTEAAIKYFLTQATASMILLMAILFNNMLSGQWTMT------------- 88
3RKO (L)  LLLMYLGWEGVGLCSYLLIGFY----YTDPKNGAAMKAFVVTRVGDVFLAFALFILYNELGTLNFR------------- 310
4HEA (T)  YPVMFIGWEGVGLASFLLIGFW----YKNPQYADSARKAFIVNRIGDLGFMLGMAILWALYGTLSIS------------- 286
4HE8 (I)  LLLMLVALEALSLPLYALATW-----RRG-QGLEAALKYFLLGALAAAFFLYGAALFYGATGSLVLG------------- 244
3RKO (N)  LASLFLGIELISLPLFGLVGYA----FRQKRSLEASIKYTILSAAASSFLLFGMALVYAQSGDLSFV------------- 288
3RKO (M)  MFLFFFFWEMMLVPMYFLIALWGHKASDGKTRITAATKFFIYTQASGLVMLIAILALVFVHYNATGVWTFNYEELLNTPM 327
4HE8 (M)  LLVFYVFFEAALIPALLMLYL-----YGGEGRTRALYTFVLFTLVGSLPMLAAVLGARLLSGSPTFL------------- 267

ND2       -NT----------TN-------------QYSSLMIMMAMAMKLGMAPFHFWVPEVTQGTPLTSGLLLLTWQKLAPISIMY 144
3RKO (L)  -EM----------VELAPAHFADGNNMLMWATLMLLGGAVGKSAQLPLQTWLADAMAGPTPVSALIHAATMVTAGVYLIA 379
4HEA (T)  -EL----------KEAMEGPLKNPDLL-ALAGLLLFLGAVGKSAQIPLMVWLPDAMAGPTPVSALIHAATMVTAGVYLIA 354
4HE8 (I)  -APGEGP------LY-------------ALALGLLLVGLGFKAALAPFHFWTPDVYQGSPTPVVLFMATSVKAAAFAALL 304
3RKO (N)  -ALGKNEP-----LL-------------LAGFGLMIVGLGFKLSLVPFHLWTPDVYQGAPAPVSTFLATASKIAIFGVVM 349
3RKO (M)  SSG----------VE-------------YLLMLGFFIAFAVKMPVVPLHGWLPDAHSQAPTAGSVDLAGILLKTAAYGLL 384
4HE8 (M)  -LEDLLAHPLQEEAA-------------FWVFLGFALAFAIKTPLFPLHAWLPPFHQENHPSGLADALGTLYKVGVFAFF 333

ND2       QI-------SPS-------L-------NVSLLLTLSILSIMAGSWGGLNQTQLRKILAYSSITHMGWMMAVLPY----N- 198
3RKO (L)  RT-------HGL-------FLMTPE--VLHLVGIVGAVTLLLAGFAALVQTDIKRVLAYSTMSQIGYMFLALGVQ---A- 439
4HEA (T)  RS-------SFL-------YSVLPD--VSYAIAVVGLLTAAYGALSAFGQTDIKKIVAYSTISQLGYMFLAAGVG---A- 414
4HE8 (I)  RV-------AAP-------P-------E--ALALLVALSVVVGNLAALAQKEAKRLLAYSSIAHAGYMALALYTG---N- 357
3RKO (N)  RL-------FLYAPVGDSEA-------IRVVLAIIAFASIIFGNLMALSQTNIKRLLGYSSISHLGYLLVALIA----LQ 411
3RKO (M)  RF-------SLP-------LFPNASAEFAPIAMWLGVIGIFYGAWMAFAQTDIKRLIAYTSVSHMGFVLIAIYTGSQLA- 449
4HE8 (M)  RFAIPLAPEGFA-------Q-------AQGLLLFLAALSALYGAWVAFAAKDFKTLLAYAGLSHMGVAALGVFSGTPEG- 398

ND2       -----PNMTILNLTIYIILTTTAFLLLNLN-----SST-------TTLLLSRTWNKLTWLTPLIPSTLLSLGGLPPLT-G 260
3RKO (L)  -----WDAAIFHLMTHAFFKALLFLASGSVILACHHEQ-------NIFKMGGLRKSIPLVYLCFLVGGAALSALPLVTAG 507
4HEA (T)  -----YWVALFHVFTHAFFKALLFLASGSVIHALGGEQ-------DVRKMGGLWKHLPQTRWHALIGALALGGLPLLS-G 481
4HE8 (I)  -----AQALGFYLLTYVLATGLAFAVLSQISP---DRV-------PLEALRGLYRKDPLLGLAFLVAMLSLLGLPPLA-G 421
3RKO (N)  TGEMSMEAVGVYLAGYLFSSLGAFGVVSLM-----SSPYRGPDADSLFSYRGLFWHRPILAAVMTVMMLSLAGIPMTL-G 485
3RKO (M)  -----YQGAVIQMIAHGLSAAGLFILCGQLYERI-HTR-------DMRMMGGLWSKMKWLPALSLFFAVATLGMPGTG-N 515
4HE8 (M)  -----AMGGLYLLAASGVYTGGLFLLAGRLYERT-GTL-------EIGRYRGLAQSAPGLAALALILFLAMVGLPGLS-G 464

ND2       FLPKWAIIEEFTKNN--SLIIPTIMATITLLNLYFYLRLIYSTSITLLPMSNNVKMKWQFEHT KPTPFLPTLIALTTLLL 338
3RKO (L)  FFSKDEILAGAMANG--HINLMVAGLVGAFMTSLYTFRMIFIVFHGKEQIHAH------AVKGVTHSLPLIVLLILSTFV 579
4HEA (T)  FWSKDAILAATLTYPFGGVGFYVGALLVAVLTAMYAMRWFVLVFLGEERGH-----HHPHEAPPVMLWPNHLLALGSVLA 556
4HE8 (I)  FWGKYLAFAEAARAG--AWGVLVLALVTSAVSAYYYLGLGLAVFARPEETP-------FRPGPPWARAAVVAAGVLLLAL 492
3RKO (N)  FIGKFYVLAVGVQAH--LWWLVGAVVVGSAIGLYYYLRVAVSLYLHAPEQPSN-------WQYSAGGIVVLISALLVLVL 556
3RKO (M)  FVGEFMILFGSFQVV---PVITVISTFGLVFASVYSLAMLHRAYFGKAKSQIASQEL--PGMSLRELFMILLLVVLLVLL 590
4HE8 (M)  FPGEFLTLLGAYKAS---PWLAALAFLSVIASAAYALTAFQKTFWEEGGSGVK-------DLAGAEWGFALLSVLALLLM 534

ND2       PISPFMLMIL* 349
3RKO (L)  GALI------* 584
4HEA (T)  GYLA------* 561
4HE8 (I)  GLLPGLVL--* 501
3RKO (N)  GVWPQPLI--* 565
3RKO (M)  GFYPQPIL--* 599
4HE8 (M)  GVFPGYFA--* 543
```

Supplementary Figure 14. Multiple sequence alignment of human ND2 with its homologs. Alignment of human ND2 to protein sequences from X-ray crystal structures used as templates to generate homology model of human ND2 by Phyre2. Each template is labeled with its PDB code (chain). Residues are shaded when sequence is in agreement for identity (black) or similarity (light grey). The groups of similar amino acids are defined as GAVLI, FYW, CM, ST, KRH, DENQ, P. Note the absence of ND2 residues that align to the N-terminal portion of the templates due to the 'short' nature of human ND2 (see text for more detail). The secondary structure prediction for human ND2 is shown schematically above the sequence.

GluN1	NMAGVF-MLVAGGIVAGIFLIFI
GluN2A	NMAGVFYMLAAAMALSLITFIW
GluN1^{insertY818}	NMAGVFYMLVAGGIVAGIFLIFI
GluN2A$^{\Delta Y822}$	NMAGVF-MLAAAMALSLITFIW
AChR α M3	**YMLFTMVFVIASIIITVIVINT**
GluN1$^{A/N\ M4}$	**YMLFTMVFVIA**GIVAGIFLIFI
GluN1$^{N/A\ M4}$	NMAGVFMLVAG**SIIITVIVINT**

Supplementary Table 1. Transmembrane domain sequence homology comparison. Sequence homology comparison between GluN1 M4, GluN2A M4, GluN1^{insertY818}, GluN2A$^{\Delta Y822}$, AChR α M3 and GluN1/AChR chimaeras. Underlined residues in GluN1 and GluN2A sequences denote identical residues. Critical methionine is in blue. Key tyrosine residue is in red. Bold denotes AChR α M3 amino acid sequence.

Template PDB code	Chain	% Identity	%Similarity
3RKO	L	12.9	34.3
4HEA	T	15.8	35.9
4HE8	I	22.9	45.2
3RKO	N	21.5	42.4
3RKO	M	15.2	33.6
4HE8	M	16.4	33.6

Supplementary Table 2. Sequence identities and similarities for ND2 and templates. Sequence identities and similarities between human ND2 and the X-ray crystal structures used as templates to generate the homology model of human ND2 by Phyre2.

Construct	Template	Primer used	Sequence (5'-3')
GluN1ΔCTD	GluN1	GluN1 Y837_STOP	CTCATTTTCATTGAGATCGCCTAAAAGCGACACAAGG
GluN1ΔATDΔCTD	GluN1ΔCTD	GluN1NdelPvu1insert1	CCTGGAGCCCTACCCCGATCGATCCCTGCTTTTTT
GluN1ΔATDΔCTD	GluN1ΔCTD	GluN1NdelPvu1insert2	ATTCAATGAGGATGGCGATCGGAAGTTTGCCAAC
GluN1ΔATDΔM4ΔCTD		GluN1 812STOP M4del	CTCACTTTTGAGAACTAGGCAGGGGTCTTCATG
GluN2A^{N1M4}	GluN2A	GluN2AswapinGluN1M4	GTGATGAGTAGCCAGCTGGACATCGATAACATGGCGGGCGTGTTCATGCTGGTGGCTGGAGGCATCGTAGCTGGGATTTTCCTCATTTTCATTGAGCACCTCTTCTACTGGAAGCTGCGCTTCTGCTTCACAGGCGTG
GluN1^{N2AM4}	GluN1	GluN1swapinGluN2AM4	CAATGCTCCTGCAACCCTCACTTTTGAGAACATGGCAGGGGTCTTCTACATGCTGGCTGCAGCCATGGCCCTCAGCCTCATCACCTTCATCTGGGAGATCGCCTACAAGCGACACAAGGATGCCCGTAGGAAGCAGATG
GluN1$^{AChr\text{-}M4}$	GluN1	AChr M3	GAATGCGACTCCCGCAGCAATGCTCCTGCAACCCTCACTTTTGAGTACATGCTATTCACCATGGTGTTCGTGATTGCAAGCATCATAATTACGGTTATCGTCATTAACACTGAGATCGCC
GluN1$^{N/A\ M4}$	GluN1	GluN1 M4/AChr M3	CTCACTTTTGAGAACATGGCAGGGGTCTTCATGCTGGTGGCTGGAAGCATCATAATTACGGTTATCGTCATTAACACTGAGATCGCCTACAAGCGACACAAGGATGCCCGTAGGAAGCAGATG
GluN1$^{A/N\ M4}$	GluN1	AChr M3/GluN1 M4	GAATGCGACTCCCGCAGCAATGCTCCTGCAACCCTCACTTTTGAGTACATGCTATTCACCATGGTGTTCGTGATTGCAGGCATCGTAGCTGGGATTTTCCTCATTTTCATTGAGATCGCCTAC
GluN1^{insertY818}	GluN1	GluN1 Tyr818Add	GAGAACATGGCAGGGGTCTTCTACATGCTGGTGGCTGGAGGCATC
GluN2A$^{\Delta Y822}$	GluN2A	GluN2A Tyr822remove	ATGGCGGGCGTGTTCATGCTGGCTGCAGCCATGGCC
GFP-ND2-TM-6-8	GFP-ND2-TM-6-11	GFPND2 223 BBS STOP	TTTCTGCTGCTGAATCTGAACAGCTGGAGATACTACGAGAGCTCCCTGGAGCCCTACCCTGACTAGAGCACCACCACCCTGCTGCTGTCT
GFP-ND2-TM-6-7	GFP-ND2-TM-6-8	GFPND2 200 BBS STOP	TGCTGCCATACAACCCAAACATGTGGAGATACTACGAGAGCTCCCTGGAGCCCTACCCTGACTAGACCATCCTGAACCTGACCATCTAC
GFP-ND2-TM-7-8	GFP-ND2	GFPND2 BamH1Met insert 175	TGGGGCGGCCTGAATGGATCCATGCTGCGCAAAATCCTG
GFP-ND2-TM-7-8	GFP-ND2	GFPND2 223 BBS STOP	TTTCTGCTGCTGAATCTGAACAGCTGGAGATACTACGAGAGCTCCCTGGAGCCCTACCCTGACTAGAGCACCACCACCCTGCTGCTGTCT
GFP-ND2-TM-8 + loop	GFP-ND2-200-300	GFPND2 E240 BBS	ACCTGGAACAAACTGACCTGGCTGTGGAGATACTACGAGAGCTCCCTGGAGCCCTACCCTGACTAGACCCCACTGATCCCAAGCACCCTG
NF-GFP-ND2-TM-6-8	GFP-ND2-TM-6-8	eGFP KO Del1-7	CCGCTAGCGCTACCGGTCGCCACCATGCTGTTCACCGGGGTGGTGCCCATC

Supplementary Table 3. Primers and templates used to create constructs.

Supplementary Note 1 – Observable currents in M4-lacking GluN1 receptors

Note that we observed currents with M4-lacking GluN1 receptors. The currents are considered *bona fide* NMDA currents as they were blocked by the competitive NMDAR antagonist D-APV. Thus, we conclude that NMDARs comprised of M4-lacking GluN1 subunits can form functional receptors. The currents were about 1-2% of the amplitude of currents generated by receptors containing full-length GluN1 subunits. As described in the Methods, our recordings of these currents were made with co-expression of PSD95. By contrast, Meddows et al[1] did not observe NMDAR currents with M4-lacking GluN1 subunits, however, they also did not co-express PSD95. PSD95 co-expression is reported to increase NMDAR currents by approximately 2-3 fold[2] by increasing cell surface levels of NMDARs and by increasing channel opening probability. Thus, the lack of PSD95 co-expression with NMDARs is the likely explanation of why Meddows et al did not detect NMDAR currents. That NMDARs comprised of M4-lacking GluN1 subunits can generate functional receptors is consistent with observations that such receptors bind glycine[3]; they can be co-immunoprecipitated with GluN2A[1]; they exist as high molecular weight complexes similar to those with full-length GluN1[1]; and they are trafficked to the cell surface at low levels[1, 4].

Supplementary References

1. Meddows E. Identification of Molecular Determinants That Are Important in the Assembly of N-Methyl-D-aspartate Receptors. *Journal of Biological Chemistry* **276**, 18795-18803 (2001).
2. Lin Y, Skeberdis VA, Francesconi A, Bennett MV, Zukin RS. Postsynaptic density protein-95 regulates NMDA channel gating and surface expression. *J Neurosci* **24**, 10138-10148 (2004).
3. Sandhu S, Grimwood S, Mortishire-Smith RJ, Whiting PJ, le Bourdelles B. Delineation of the structural determinants of the N-methyl-D-aspartate receptor glycine binding site. *J Neurochem* **72**, 1694-1698 (1999).
4. Horak M, Chang K, Wenthold RJ. Masking of the Endoplasmic Reticulum Retention Signals during Assembly of the NMDA Receptor. *Journal of Neuroscience* **28**, 3500-3509 (2008).
